# Longitudinal Dataset of Net-load, PV Production and Solar Irradiation from Madeira Island, Portugal

**DOI:** 10.1038/s41597-025-06118-x

**Published:** 2025-11-28

**Authors:** Lucas Pereira, Diogo Monteiro, Frederick Apina, Sabrina Scuri, Mary Barreto, Filipe Quintal, Hugo Morais

**Affiliations:** 1 Interactive Technologies Institute, LARSyS, 1049-001 Lisbon, Portugal; 2 Instituto Superior Técnico (IST), University of Lisbon, 1049-001 Lisbon, Portugal; 3 Instituto de Engenharia de Sistemas e Computadores - Investigação e Desenvolvimento, 1049-001 Lisbon, Portugal; 4 University of Madeira, Faculty of Exact Sciences and Engineering, 9020-105 Funchal, Portugal; 5 Design Department, Politecnico di Milano, 20158 Milan, Italy

**Keywords:** Energy science and technology, Energy infrastructure

## Abstract

This paper presents the PTProsumer dataset, a high-resolution dataset of photovoltaic (PV) production and net-load measurements collected from 24 prosumers - entities that both produce and consume electricity, including households and small commercial buildings - on Madeira Island, Portugal. The dataset covers monitoring periods ranging from 3 months to 5 years, with measurements sampled at a 1-second resolution, resulting in approximately 3.89 billion data points. PV production and electricity consumption are provided as separate signals, enabling detailed analysis of local energy flows. The dataset also includes 1-minute resolution solar irradiation estimates from the Copernicus Atmosphere Monitoring Service. Files are provided in CSV format, organized by year, and are accompanied by a public code repository with examples for data processing. This dataset supports research in forecasting, flexibility management, grid resilience, and energy community modeling, contributing to the growing need for open-access, long-term energy datasets.

## Background & Summary

The global energy landscape is undergoing a profound transformation with the rise of prosumers —consumers who also produce energy, most notably through residential and small commercial Solar Photovoltaic (PV) systems. By 2024, Europe’s total installed solar capacity reached 338 GW, up from 272.5 GW in 2023 and 207 GW in 2022^1^. However, despite this overall growth, residential rooftop solar installations declined. New home installations dropped by nearly 5 GW compared to the previous year, totaling only 12.8 GW in 2024^1^. This slowdown is attributed not only to reduced financial incentives and grid connection delays, but also to increasing regulatory uncertainty—including limitations on self-consumption and the export of surplus generation—which complicate the economic viability for individual households^1,2^.

These evolving regulatory and market conditions highlight the urgent need to better manage distributed assets, ensuring that prosumers can maximize the value of their generation and continue contributing to local energy resilience and flexibility. However, this demands differentiated management strategies: targeting flexible demand response mechanisms for highly variable, high-demand sites, while optimizing storage solutions or export mechanisms for prosumers with frequent surplus generation, e.g.,^3–5^.

This emerging reality highlights the importance of high-quality prosumer datasets, particularly those with long-term monitoring to accurately capture seasonal variations in consumption and generation patterns. Although numerous public datasets focus on household electricity consumption, as surveyed in several review papers, e.g.,^6,7^, there remains a shortage of comprehensive real-world datasets that include both PV generation and net-load measurements.

Among the few datasets offering both consumption and PV production data, PecanStreet is the most widely known^8^. It provides high-resolution (up to 1-second) household consumption, solar generation, and appliance-level data for thousands of U.S. households. Data collection started in 2012 and continues today, with multi-year records available for many PV sites. A consultation of the latest available metadata at the time of writing revealed that 551 of 2035 sites have both consumption and production data. However, full access is restricted since researchers must sign a data use agreement, and some datasets require payment. A smaller, free subset is available for academic research. The Smart* SunDance dataset provides hourly net-metered consumption, solar generation, and weather data for 100 solar sites across North America, covering a full year from January 2015 to January 2016, and has been utilized for studies on solar forecasting and energy modeling^9^. As of this writing, the SunDance dataset can be downloaded from the UMass Trace Repository (https://traces.cs.umass.edu/docs/traces/smartstar/#sundance-dataset, Accessed: Jul. 30, 2025). The StoreNet dataset provides detailed energy data from 20 households in Ireland, collected under the StoreNet project^10^. It includes power and energy measurements (consumption, PV generation, grid import/export, battery charging/discharging, and state of charge) along with local weather data at 1-minute resolution for the year 2020. For 10 houses, there are also PV production measurements, from systems with capacities ranging between 2 and 2.2 kWp. The HEMStoEC database offers detailed monitoring data over more than three years (January 2020 to February 2023) for four houses in southern Portugal, including high-frequency (1-second and 1-minute) records of consumption, PV and battery data, indoor climate conditions, and device operation, with synchronized datasets available at 5-minute intervals and detailed metadata on data gaps and interpolation periods^11^. Finally, a very recent dataset is from a residential dwelling in Estonia, providing one year (2023) of 10-second resolution PV generation and power flow measurements^12^.

To help address this lack of representative and reusable data, we present PTProsumer^13^, a dataset featuring measurements of net-load and PV production at 1-second resolution for 24 prosumers located exclusively on Madeira Island, Portugal. The data collection spans monitoring periods ranging from approximately 3 months to 5 years per prosumer. PTProsumer was collected within the scope of the H2020 Smart Island Energy Systems (SMILE) project (https://cordis.europa.eu/project/id/731249, Accessed Jul. 30, 2025), an initiative focused on demonstrating smart grid solutions for isolated energy systems, with demonstrators in three European islands, namely Madeira, Orkney, and Samsø. Additionally, the dataset was extended with irradiation data from the Copernicus Atmosphere Monitoring Service (CAMS), including Global Horizontal Irradiance (GHI), and Diffuse Horizontal Irradiance (DHI), providing a more complete characterization of the solar resource conditions during the monitoring period.

For a direct comparison among the mentioned datasets, Table 1 summarizes their key attributes, including location, number of sites, type of prosumers, dataset duration, sampling rate, and the available measurements. Among the six datasets compared, PTProsumer distinguishes itself through its combination of high-frequency (1-second) measurements, building types (including apartments, houses, and commercial spaces), and extended monitoring periods ranging from 3 months to 5 years. Covering data from 2018 to 2024 and including a wide array of electrical parameters along with solar irradiance, it complements existing datasets by offering a uniquely rich and versatile resource for studying European prosumers in real-world conditions.

**Table 1:** Summary of key characteristics of the reviewed prosumer energy datasets.

Name	Location	Sites	Types	Duration	Period	Sampling	Measurements
Pecan Street	USA	>1000^a^	A, H	Multi-year	2012–today	1-second	P, W
SunDance	USA	100	H	1-year	2015–2016	1-hour	P, W
StoreNet	Ireland	20^b^	H	1-year	2020	1-minute	P, E, W
N/A	Estonia	1	H	1-year	2023	10-second	V, P, PF
HEMStoEC	Portugal	4	H	>3-year	2020–2023	1-second	I, V, P, Q, S, F, PF, E
PTProsumer	Portugal	24	A, H, O, R	3-month - 5-year	2018–2024	1-second	I, V, P, Q, S, PF, F, Ir

Types: Apartment (A), Houses (H), Offices (O), Restaurants (R).

Measurements: Current (I), Voltage (V), Active Power (P), Reactive Power (Q), Apparent Power (S), Solar Irradiance (Ir), Frequency (F), Power Factor (PF), Energy (E), Weather (W).

^a^40 homes (~1 year, one-second resolution) are available for free to qualifying university researchers.

^b^Only 10 houses contain both consumption and PV production.

Given its high temporal resolution and long-term coverage, this dataset holds significant potential for reuse across research domains that rely on smart meter data, such as demand forecasting, flexibility characterization, PV self-consumption optimization, and energy community modeling^14–16^. The fine-grained resolution enables detailed analysis of consumption and PV production dynamics, while also allowing for flexible aggregation to coarser resolutions (e.g., 15-minute and hourly), which is often required in practical applications. The extended monitoring duration further supports longitudinal analyses of prosumer behavior and seasonal patterns. The dataset’s relevance has already been demonstrated through several scientific outputs, including techno-economic analyses of PV-BESS systems^17,18^, storage sizing^19,20^, explainable AI in PV production forecasting^21^, and federated learning approaches for flexibility markets^22^.

This paper thoroughly describes how the PTProsumer dataset was collected, including detailed information on post-processing and organizing the data to form the dataset. This paper also analyzes the data quality and provides instructions for its reuse.

## Methods

### Data Collection Setup: Net-Load and PV Production Measurements

The data collection for Net-Load and PV production was carried out using Carlo Gavazzi (CG) smart meters. Specifically, two CG meters were DIN-mounted in the main breaker box: one for measuring grid consumption and another for PV production. Three different models were used, selected based on installation requirements.

- EM111: A compact model (17.5 mm wide) that supports currents up to 45A and a maximum cable cross-section of 6mm^2^. This model was primarily used for PV production measurements.
- EM112: Similar to the EM111 but slightly wider (35 mm), supporting currents up to 100A and cable cross-sections up to 25mm^2^. This device was mainly used for consumption measurements.
- EM340: A three-phase smart meter supporting currents up to 100A and cable cross-sections up to 25mm^2^, used for three-phase installations.

**Fig. 1:**
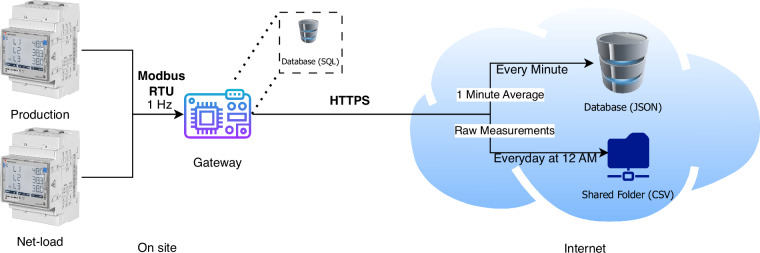
Overview of the net-load and PV production data collection setup for a three-phase installation.

Figure 1 illustrates the main components of the data collection setup. The CG meters were installed in the main breaker box, and a Raspberry Pi 3 gateway was connected to them via a serial port using the RS485 communication protocol. The gateway sequentially requested the latest measurements from each meter at a configurable scanning interval, set to one second in this setup. The timestamp of the request was shared between the two meters, ensuring that records from a single scan had the same timestamp.

The collected samples were stored in a relational database on the gateway and uploaded every minute to an online database server, providing real-time access to consumption data^23^. Additionally, at 12 AM daily, a Comma Separated Values (CSV) file containing the day’s readings for each CG meter was uploaded to a shared folder. After successful uploads, the local database was cleared to minimize storage footprint.

### Data Collection Setup: Irradiation Measurements

Solar irradiation data was obtained using the CAMS service^24^, which provides high-resolution estimates under both clear-sky and all-sky conditions. The clear-sky irradiation values are produced using the CAMS global forecasting system, which incorporates atmospheric components such as aerosol optical depth, ozone concentration, and water vapor content. These components are essential in the CAMS methodology, as they influence the scattering and absorption of solar radiation in the atmosphere, thereby affecting the irradiance reaching the surface. All-sky irradiation estimates, on the other hand, are derived from satellite imagery within the field-of-view of the Meteosat Second Generation (MSG) and Himawari satellites. Irradiation data was retrieved at a 1-minute temporal resolution, aligned with the net-load and PV production measurements, and geolocated using the approximate coordinates of each prosumer—omitted here for privacy.

### Deployment

The recruitment of prosumers and the deployment of the data collection setup occurred in three phases: i) between March and June of 2018, ii) in October and November of 2020, and iii) between February and March 2020. The dataset comprises 24 monitored sites, representing a diverse set of prosumers, including residential houses (17), apartments (4), an office, a restaurant, and a supplier.

Ethical approval for the data collection activities was granted by the SMILE project’s Ethics Board, established at Month 3 of the project to oversee compliance with ethical and privacy requirements defined under Work Package 10 (“Ethics Requirements”). The Ethics Board reviewed and approved the procedures and consent forms used across the three project demonstrators and continuously monitored the project to ensure participant protection and data privacy. All participants signed the informed consent prior to the installation of monitoring equipment and the start of data collection. Ethical requirements and approvals are documented in the project’s internal deliverables D10.1 (H-Requirement No. 1) and D10.2 (POPD - Requirement No. 2).

In each site, the consumption meter was installed immediately downstream of the utility meter, measuring only the energy drawn from the grid (net-load), not the total household consumption. On the other hand, the PV meter was installed directly upstream of the PV inverter, capturing the total PV generation before any self-consumption or grid export occurs.

Table 2 provides detailed information on each monitored site, including the type of prosumer, connection characteristics, monitoring periods, and data completeness percentages. The monitored sites vary in grid connection phases (single-phase and three-phase) and peak power capacities, with grid-side peak power ranging from 3.45 kW to 20.7 kW and production-side peak power from 0.39 kW to 4.32 kW. The data collection periods span from March 2018 to December 2023, with most sites achieving high data completeness, typically exceeding 90%. However, certain locations, particularly apartments, exhibit lower completion rates, with some below 50%, due to interruptions in data collection as a result of hardware connectivity issues. In many apartments, the main electrical panels were located near the entrance, where the Wi-Fi signal strength was insufficient for reliable transmission. Additionally, changes to Wi-Fi credentials by residents, without notice to the research team, required on-site visits to reestablish connectivity. In 23 of the 25 sites, the completion rates of Net-load and PV production are very similar. The only exception is PR_21 (Net-load: 92.4%, PV: 73.98%), due to technical issues with the PV smart meter. Net-load data is available as single-phase for 20 sites and three-phase for 4 sites, while production data is available as single-phase for 22 sites and three-phase for 2 sites.

**Table 2:** Overview of monitored prosumers, including installation characteristics, monitoring periods, and data completeness.

ID	Type	Phases		Peak Power		Collection Period		Percent Complete	
		Cons.	Prod.	Cons.	Prod.	Start	End	Net-load	Prod.
PR_01	Office	1	1	10.35	1.50	29/03/2018	21/02/2022	85.13	85.13
PR_02	House	3	1	10.35	2.70	01/05/2018	21/02/2022	94.94	95.04
PR_03	Restaurant	3	3	20.70	3.92	25/05/2018	20/05/2022	96.75	97.46
PR_04	House	1	1	6.90	1.25	29/05/2018	24/01/2020	99.20	99.28
PR_05	Apartment	1	1	6.90	0.50	06/06/2018	13/03/2023	95.53	95.55
PR_06	House	1	1	3.45	0.75	09/06/2018	21/02/2022	98.63	99.61
PR_07	House	1	1	6.90	0.39	19/06/2018	27/03/2021	99.40	99.40
PR_08	House	1	1	6.90	1.50	20/06/2018	20/09/2018	98.20	98.20
PR_09	House	1	1	6.90	1.50^a^	23/06/2018	31/12/2023	87.69	87.68
PR_10	House	1	1	10.35	0.50	28/06/2018	24/06/2020	97.88	97.88
PR_11	House	3	1	10.35	1.50^b^	29/06/2018	16/07/2022	88.02	90.93
PR_12	House	1	1	6.90	0.50	29/06/2018	21/02/2022	95.91	95.94
PR_13	House	1	1	6.90	0.50	29/06/2018	01/02/2021	90.09	90.09
PR_14	Apartment	1	1	10.35	0.50	10/07/2018	31/12/2023	47.61	47.63
PR_15	Apartment	1	1	6.90	0.50	10/07/2018	03/03/2023	46.89	38.41
PR_16	House	1	1	6.90	0.50	11/07/2018	02/07/2021	74.97	74.98
PR_17	Apartment	1	1	6.90	0.50	21/07/2018	25/03/2023	27.25	27.26
PR_18	House	1	1	3.45	1.00	17/10/2019	22/08/2021	92.45	92.46
PR_19	House	1	1	6.90	1.88	22/11/2019	12/06/2021	84.11	84.11
PR_20	House	1	1	10.35	0.75	22/11/2019	21/12/2023	98.70	99.47
PR_21	House	1	1	10.35	1.74	19/12/2019	10/06/2021	92.41	73.98
PR_22	Supplier	3	3	10.80	4.32	15/02/2020	18/11/2022	87.34	87.70
PR_23	House	1	1	6.90	1.68	27/02/2020	31/12/2020	95.98	96.09
PR_24	House	1	1	6.90	1.00	13/03/2020	27/11/2022	97.49	98.25

^a^This prosumer upgraded the installation to 2.25kWp on 13/12/2019.

^b^This prosumer upgraded the installation to 4.5kWp on 07/08/2020.

### Data Post-Processing

The PV production data was post-processed to account for small nighttime reverse currents, mitigate measurement noise from the inverter and sensors, and correct for power consumption by the monitoring equipment. These factors can introduce slight deviations in the form of small non-zero values, despite the physical impossibility of solar generation in the absence of sunlight. Using the Astral (v3.2) Python package to determine sunrise and sunset times based on each prosumer’s approximate geographical coordinates, recorded PV power values outside the daylight periods were assumed to be measurement artifacts and were consequently set to zero.

In contrast, net-load data did not require post-processing, as grid import/export measurements are generally more stable and less affected by sensor noise. Additionally, reverse currents do not impact net-load since they are naturally accounted for in grid import/export measurements, and the power consumption of monitoring equipment is negligible compared to total household consumption.

## Data Records

**Fig. 2:**
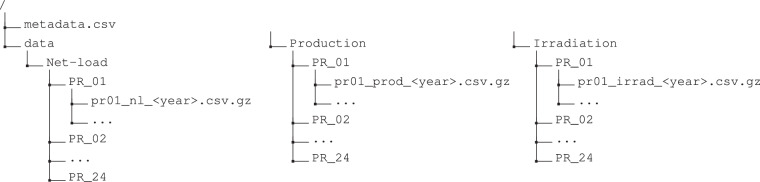
Underlying folder and file organization of PTProsumer Dataset.

The PTProsumer dataset is made available individually for each monitored prosumer, and all the data files are in CSV format. The data is available on the Open Science Framework (OSF) data repository^13^. Figure 2 shows an overview of the underlying organization of the PTProsumer dataset. The following subsections describe the contents of the different files.

### Metadata

A CSV version of Table 2 is available in the datasets’ root folder in a file named metadata.csv. This file contains 24 rows. The columns are described in Table 3.

**Table 3:** Description of the metadata.csv file.

Column	Description	Units
ID	Prosumer ID	—
Type	Type of prosumer: Apartment, House, Office or Restaurant	—
Phases_Cons	Number of phases in the consumption installation	—
Phases_Prod	Number of phases in the production installation	—
Peak_Power_Cons	Peak power on the consumption installation	*kW*
Peak_Power_Prod	Peak power on the production installation	*kW*
Collection_Start	Date when the data collection started	*date*
Collection_End	Date when the data collection ended	*date*
Complete_Net-Load	Ratio of net-load data points to the total expected points over the monitoring period	*%*
Complete_Production	Ratio of production data points to the total expected points over the monitoring period	*%*

### Net-Load and PV Production Measurements

The consumption measurements are available in individual files for each year in the Net-Load folder. The measurement files, named pr<ID>_nl_<year>.csv, are available in the root folder of each prosumer (PR_<ID>). The production files follow the same organization but instead are stored under the Production folder and titled pr<ID>_prod_<year>.csv. The columns of the CSV files are described in Table 4.

**Table 4:** Description of the net-load (pr<ID>_nl_<year>.csv) and production (pr<ID>_prod_<year>.csv) files.

Column	Description	Units
timestamp	Timestamp (YYYY-MM-DD HH:MM:SS) when the record was collected (UTC)	*datetime*
voltage_P1	Voltage in Phase 1	*Volt*
voltage_P2	Voltage in Phase 2 (if available)	*Volt*
voltage_P3	Voltage in Phase 3 (if available)	*Volt*
current_P1	Current in Phase 1	*Amp*
current_P2	Current in Phase 2 (if available)	*Amp*
current_P3	Current in Phase 3 (if available)	*Amp*
active_power_P1	Active Power in Phase 1	*Watt*
active_power_P2	Active Power in Phase 2 (if available)	*Watt*
active_power_P3	Active Power in Phase 3 (if available)	*Watt*
apparent_power_P1	Reactive Power in Phase 1	*VA*
apparent_power_P2	Reactive Power in Phase 2 (if available)	*VA*
apparent_power_P3	Reactive Power in Phase 3 (if available)	*VA*
reactive_power_P1	Reactive Power in Phase 1	*VAR*
reactive_power_P2	Reactive Power in Phase 2 (if available)	*VAR*
reactive_power_P3	Reactive Power in Phase 3 (if available)	*VAR*
power_factor_P1	Power Factor in Phase 1	—
power_factor_P2	Power Factor in Phase 2 (if available)	—
power_factor_P3	Power Factor in Phase 3 (if available)	—
frequency	Average System Frequency	*Hz*

### Irradiation Measurements

The solar irradiation measurements are available in individual files for each year in the Irradiation folder. The measurement files, named pr<ID>_irrad_<year>.csv, are available in the root folder of each prosumer (PR_<ID>). The columns of the CSV files are described in Table 5.

**Table 5:** Description of the irradiation (pr<ID>_rad_<year>.csv) files.

Column	Description	Units
timestamp	Timestamp (YYYY-MM-DD HH:MM:SS) when the observation period ended (UTC)	*datetime*
TOA	Irradiation on a horizontal plane at the top of the atmosphere	*Wh* *m*^−2^
Clear sky GHI	Clear sky global irradiation on a horizontal plane at ground level	*Wh* *m*^−2^
Clear sky BHI	Clear sky beam irradiation on a horizontal plane at ground level	*Wh* *m*^−2^
Clear sky DHI	Clear sky diffuse irradiation on a horizontal plane at ground level	*Wh* *m*^−2^
Clear sky BNI	Clear sky beam irradiation on the mobile plane following the sun at normal incidence	*Wh* *m*^−2^
GHI	Global irradiation on a horizontal plane at ground level	*Wh* *m*^−2^
BHI	Beam irradiation on a horizontal plane at ground level	*Wh* *m*^−2^
DHI	Diffuse irradiation on a horizontal plane at ground level	*Wh* *m*^−2^
BNI	Beam irradiation on mobile plane following the sun at normal incidence	*Wh* *m*^−2^

## Technical Validation

**Fig. 3:**
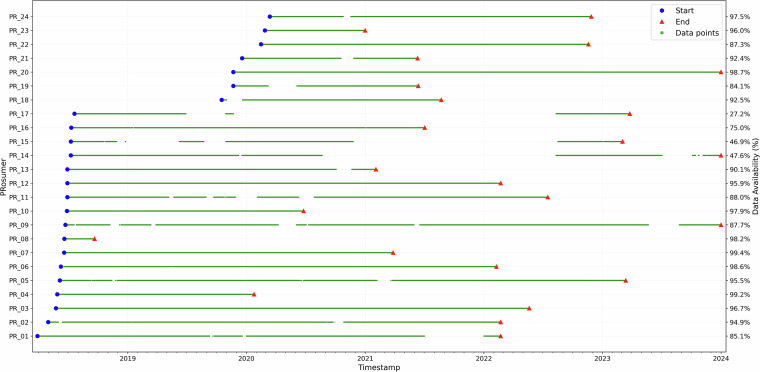
PTProsumer Net-load Data Availability. Gaps in the line represent an area where data was unavailable for more than a quarter of a day.

**Fig. 4:**
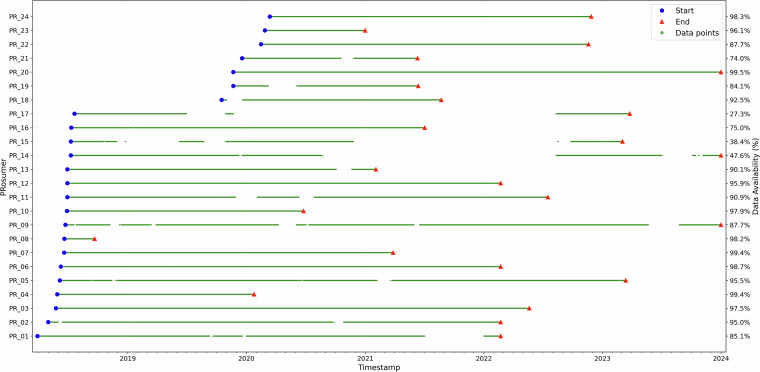
PTProsumer PV Production Data Availability. Gaps in the line represent an area where data was unavailable for more than a quarter of a day.

The net-load and PV production data availability for all PTProsumer sites can be seen in Figs. 3 and 4. The gaps indicate periods when the data were unavailable. The vertical right axis of both figures shows the data availability (%), calculated as the ratio of available data points to the total expected data points over the period each prosumer was monitored.

The monitoring campaign had varying durations and data completeness levels. The earliest installation began on March 29, 2018, while the latest started on March 13, 2020, with monitoring periods ranging from just a few months to nearly 5.5 years. Some sites, such as Site 9 (House) and Site 14 (Apartment), were monitored until December 31, 2023, providing some of the longest datasets. In contrast, Site 8 (House) was active for only three months, and Site 23 (House) for just ten months, limiting their ability to capture long-term trends.

A key observation is that long monitoring periods do not necessarily imply high data completeness. Site 17 (Apartment), despite being monitored for almost five years, suffered from significant data loss, with a completeness of just 27.2%, indicating extended periods of missing data. Similarly, Site 14 (Apartment), which also had one of the longest monitoring durations, maintained only 47.6% completeness. Conversely, some short-duration sites achieved nearly uninterrupted data collection. Site 8 (House), monitored for only three months, maintained a 98.20% completeness rate, ensuring a high-quality dataset despite its brevity. Site 7 (House), with a monitoring period of just under three years, achieved 99.40% completeness, demonstrating the stability and reliability of its data stream. Among the longest monitored sites, Site 3 (Restaurant) and Site 20 (House) stand out, as both provided four to five years of data with nearly 99% completeness.

**Fig. 5:**
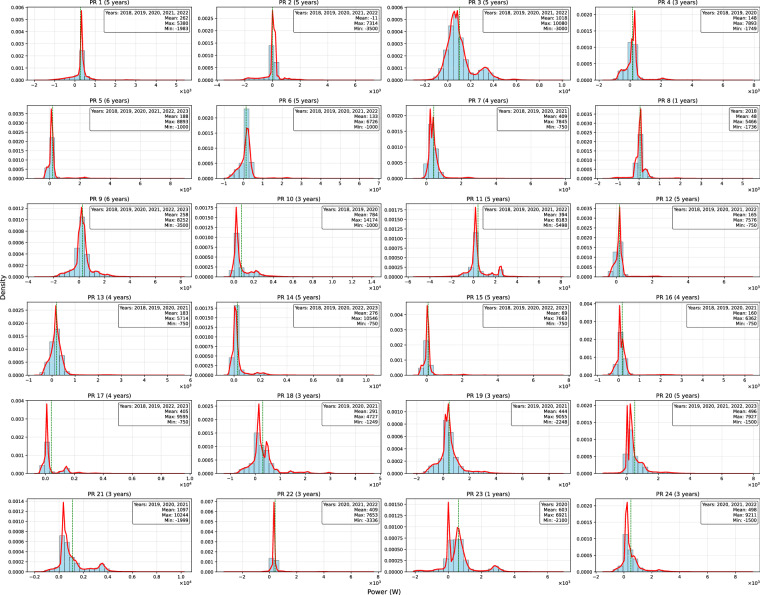
PTProsumer Net-load data distribution represented through histograms. Mean, minimum, and maximum values are indicated to support the interpretation of central tendency and range.

**Fig. 6:**
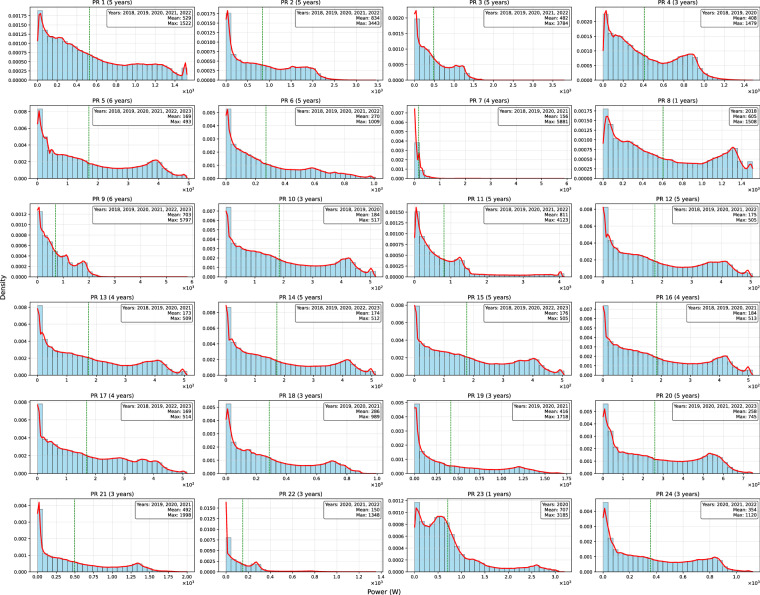
PTProsumer PV Power distribution represented through histograms. Mean maximum values are indicated to support the interpretation of central tendency and range.

**Fig. 7:**
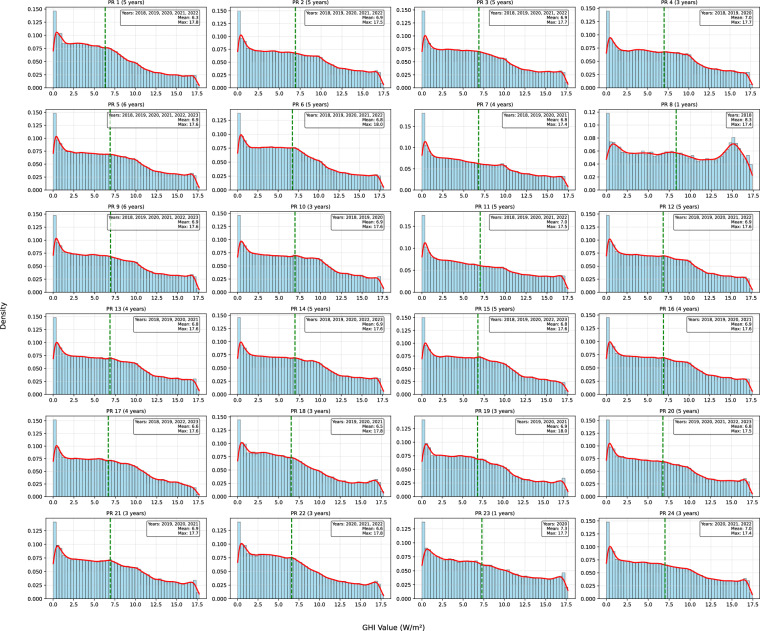
PT Prosumer Irradiation distribution represented through histograms. Mean maximum values are indicated to support the interpretation of central tendency and range.

To further understand the characteristics of each site, the histograms in Figs. 5, 6, 7 provide a statistical overview of the data by illustrating the distribution of key variables (active power for net-load and PV production, and GHI for irradiation). They aim to illustrate the distribution of values, enabling the identification of central tendencies, dispersion, and potential anomalies within the collected data. Each histogram is complemented by key statistical values such as Mean, Minimum, and Maximum, which support the interpretation of the data’s range and central tendency.

The net-load statistics of the 24 prosumers reveal a highly heterogeneous group, with substantial differences in consumption and generation dynamics. Several prosumers (e.g., PR_2, PR_8, PR_15) exhibit frequent periods of net-export, as indicated by negative or near-zero 25th percentile values. In contrast, others, such as PR_21 and PR_23, maintain consistently positive net-loads across all quartiles, reflecting a strong and steady demand profile. Prosumers like PR_3, PR_10, and PR_21 reach maximum net-loads exceeding 10 kW. High variability is also evident, particularly for PR_3, PR_21, and PR_23, where standard deviations approach or exceed 1 kW, while more stable profiles are seen in prosumers like PR_5 and PR_7.

The PV production profiles in the dataset demonstrate considerable diversity, with mean values ranging from approximately 150 W to over 830 W across different prosumers. Several sites, such as PR_2, PR_9, PR_11, and PR_23, recorded maximum generation peaks well above 3000 W, indicating substantial PV system sizes. The standard deviation is consistently high relative to the mean for many sites, indicating significant intra-day and inter-day variability. Percentile values further reveal that for most prosumers, production is skewed, with median values (50th percentile) often much lower than the maximum observed outputs. This highlights that, although large generation events occur, they are not dominant throughout the measurement period.

The Global Horizontal Irradiance (GHI) across the 24 prosumer sites is relatively consistent, with mean values typically between 6.3 and 7.3 W/m^2^, indicating uniform solar resource availability across the region. The standard deviation ranges between 4.5 and 5.2 W/m^2^, reflecting natural daily and seasonal solar variability. Overall, the GHI data show that differences in PV production between prosumers are more likely due to system-specific factors such as panel make and model, inverter efficiency, installation orientation and tilt, shading from nearby objects, maintenance practices, or variations in monitoring equipment, rather than significant differences in solar irradiance.

## Usage Notes

### Decompressing and Reading Files

The PTProsumer dataset^13^ is made available in CSV format, which is usable in most scientific computing packages, e.g., Python (pandas/numpy), MATLAB (readmatrix), and R (read.csv). However, due to the large size of the raw data, it was decided to store the files in compressed format using gzip. This is a very common protocol, and the files can be easily unzipped using software such as 7-Zip, WinRAR, or the built-in gunzip utility on Linux and macOS systems. Alternatively, most scientific programming packages offer tools to decompress and read the CSV files directly; for example, in Python, libraries such as pandas, gzip, and zipfile can be used. Given the volume of data, parallel processing can also be beneficial for faster data handling. We provide example Python3 scripts and guidance for both sequential and parallel data processing in the available source code.

### Missing Data

While there is some missing data in every PTProsumer, we deliberately did not clean or strip any data. This allows us to retain the data from its raw form as closely as possible. Furthermore, this leaves room for the dataset users to apply their preferred data-cleaning and filling methods. For example, in the case of PV production, missing data can be completed leveraging irradiation data and the information about the installed PV capacity on each site. Several libraries exist for this effect, including *pvlib python*^25^. As for the net-load, several methods can be applied depending on the number of missing points. Such strategies can vary from quick fixes such as forward/backward filling or interpolation (linear or weighted) to more advanced techniques using forecasting algorithms, such as the Auto-Regressive Integrated Moving Average (ARIMA)^26^.

### Handling Timestamps

This dataset was collected in Madeira Island in Portugal, and during the data collection, two different time zones were observed: Western European Time (WET) and Western European Summer Time (WEST), following the region’s standard daylight saving time changes. However, to simplify the dataset handling, it was decided to ignore the timezone by representing the timestamps in Coordinated Universal Time (UTC). To state more precisely, when DST starts (clocks move forward), local time skips one hour (e.g., from 1:00 AM to 2:00 AM), but since time is recorded in UTC, no data is lost—timestamps simply continue uninterrupted (e.g., 00:59:59 UTC  → 01:00:00 UTC). Similarly, when DST ends (clocks move back), local time repeats an hour (e.g., 2:00 AM occurs twice), but again, because of using UTC, there is no duplication—timestamps continue progressing forward without repeating. If needed, local time can be recovered from UTC using the known time zone of Madeira (WET/WEST) and standard libraries. For example, using Python pytz, setting the timezone to Atlantic/Madeira.

### Data Collection During COVID-19

Data collection overlapped with the COVID-19 pandemic, particularly during the first strict lockdown period in Madeira Island, which lasted from March 19 to May 3, 2020. This context may have led to atypical consumption behaviors, especially due to stay-at-home orders and the closure of commercial activities.

### Dataset Limitations

While the dataset captures detailed net-load and PV production measurements from each prosumer, it does not include contextual information about the physical installation conditions of the PV systems. Specifically, no data were collected regarding panel orientation, tilt, or potential sources of partial shading such as nearby buildings, trees, or obstructions. As a result, variations in PV output caused by suboptimal placement or shadowing are not accounted for. This limitation should be considered when interpreting the production data or using it to develop or validate models that assume ideal or standardized PV operating conditions.

Likewise, despite the fact that the dataset includes GHI estimates obtained from the CAMS radiation service, no on-site pyranometer measurements were available to validate the modeled values. As such, potential discrepancies between modeled and ground-based radiation values are not quantified. Future deployments may incorporate on-site solar irradiation measurements to improve the accuracy and validation of the irradiance data. Additionally, future work could include comparisons with alternative forecasting services, such as Forecast.Solar, to assess the consistency and potential biases across different sources of solar irradiation estimates.

Table 6 provides a brief description of the available source code.

**Table 6:** Brief description of the available source code.

File	Description
decompress_data.py	Code to decompress all .gz files in the dataset and save the extracted data as CSV files.
analyze_data.ipynb	Code to analyze and validate Net-Load, PV Production, and Radiation data. Generates statistics and visualizations based on the defined data sampling/resolution.

## Data Availability

The dataset^13^ is publicly available in its own repository on the Open Science Framework at https://osf.io/9xs3a/.

## Code availability

The Python3 code to reproduce the examples presented in this paper is available on the dataset repository^13^.

## References

[1] SolarPower Europe, *European Market Outlook for Solar Power 2024–2028*, Brussels, Belgium: SolarPower Europe, (2024). Available at: https://www.solarpowereurope.org/insights/outlooks/eu-market-outlook-for-solar-power-2024-2028 (Accessed: 2025).

[2] International Energy Agency - Photovoltaic Power Systems Program, *PVPS Annual Report 2024: Country Updates*, NSW, Australia: International Energy Agency, Apr. (2025). Available at: https://iea-pvps.org/wp-content/uploads/2025/04/PVPS_Annual_Report_2024_Country_Updates.pdf (Accessed: 2025).

[3] Pamfile L. V. & Proscanu, M. E. Energy Transition: How Solar PV Would Shape the Final Household Electricity Consumption. An Economic Analysis on Sizing, Integration and Risk of Prosumer Energy Systems. in *Rethinking Business for Sustainable Leadership in a VUCA World*, edited by M. Busu, pp. 245–262, Springer Nature Switzerland, Cham, 10.1007/978-3-031-50208-8_16 (2024).

[4] Díaz-Bello, D., Vargas-Salgado, C., Alcázar-Ortega, M. & Gómez-Navarro, T. Demand response of prosumers integrating storage system for optimizing grid-connected photovoltaics through time-pricing. *Journal of Energy Storage* **88**, 111536, 10.1016/j.est.2024.111536 (2024).

[5] Benalcazar, P., Kalka, M. & Kamiński, J. From consumer to prosumer: A model-based analysis of costs and benefits of grid-connected residential PV-battery systems. *Energy Policy* **191**, 114167, 10.1016/j.enpol.2024.114167 (2024).

[6] Pereira, L. & Nunes, N. Performance evaluation in non-intrusive load monitoring: datasets, metrics, and tools-a review. *WIREs Data Mining and Knowledge Discovery* **8**(6), e1265, 10.1002/widm.1265 (2018).

[7] Haben, S., Arora, S., Giasemidis, G., Voss, M. & Vukadinović Greetham, D. Review of low voltage load forecasting: methods, applications, and recommendations. *Applied Energy* **304**, 117798, 10.1016/j.apenergy.2021.117798 (2021).

[8] Pecan Street Project, Inc., *Pecan Street Grid Demonstration Program. Final Technology Performance Report*, Austin, TX (United States), 10.2172/1172297 (2015).

[9] Chen, D. & Irwin, D. SunDance: black-box behind-the-meter solar disaggregation. in *Proceedings of the Eighth International Conference on Future Energy Systems*, pp. 45–55, 10.1145/3077839.3077848 (Association for Computing Machinery, New York, NY, USA, May 2017).

[10] Trivedi, R., Bahloul, M., Saif, A., Patra, S. & Khadem, S. Comprehensive dataset on electrical load profiles for energy community in Ireland. *Scientific Data* **11**(1), 621, 10.1038/s41597-024-03454-2 (2024). 38866794 10.1038/s41597-024-03454-2 PMC11169232

[11] Ruano, A. & da Graça Ruano, M. From home energy management systems to energy communities: methods and data. *Scientific Data* **11**(1), 346, 10.1038/s41597-024-03184-5 (2024). 38582775 10.1038/s41597-024-03184-5 PMC10998857

[12] Hasan, S., Blinov, A., Chub, A. & Vinnikov, D. Solar PV generation and consumption dataset of an Estonian residential dwelling. *Scientific Data* **12**(1), 481, 10.1038/s41597-025-04747-w (2025). 40121221 10.1038/s41597-025-04747-w PMC11929768

[13] L. Pereira, D. R. P. Monteiro, and F. Apina, *Longitudinal Dataset of Net-load, PV Production and Solar Irradiation from Madeira Island, Portugal*, Available at: 10.17605/OSF.IO/9XS3A, OSF (2025). 10.1038/s41597-025-06118-x PMC12675649 41315408

[14] Wang, Y., Chen, Q., Hong, T. & Kang, C. Review of smart meter data analytics: applications, methodologies, and challenges. *IEEE Transactions on Smart Grid* **10**(3), 3125–3148, 10.1109/TSG.2018.2818167 (2019).

[15] Völker, B., Reinhardt, A., Faustine, A. & Pereira, L. Watt’s up at home? Smart meter data analytics from a consumer-centric perspective. *Energies* **14**(3), 719, 10.3390/en14030719 (2021).

[16] Faia, R., Goncalves, C., Gomes, L. & Vale, Z. Dataset of an energy community with prosumer consumption, photovoltaic generation, battery storage, and electric vehicles. *Data in Brief* **48**, 109218, 10.1016/j.dib.2023.109218 (2023). 37383810 10.1016/j.dib.2023.109218 PMC10293983

[17] Pereira, L., Cavaleiro, J. & Barros, L. Economic assessment of solar-powered residential battery energy storage systems: the case of Madeira Island. Portugal. *Applied Sciences* **10**(20), 7366 (2020).

[18] Pereira, L., Cavaleiro, J. & Morais, H. Understanding the role of solar PV and battery energy storage in a snack bar: a case study in Madeira Island, in *2023 IEEE 21st International Conference on Industrial Informatics (INDIN)*, pp. 1–7, 10.1109/INDIN51400.2023.10218295 (IEEE, 2023).

[19] Hashmi, M. U., Pereira, L., & Bušić, A. Energy storage in Madeira, Portugal: co-optimizing for arbitrage, self-sufficiency, peak shaving and energy backup. in *2019 IEEE Milan PowerTech*, pp. 1–6, 10.1109/PTC.2019.8810531 (IEEE, 2019).

[20] Hashmi, M. U., Cavaleiro, J., Pereira, L. & Bušić, A. Sizing and profitability of energy storage for prosumers in Madeira, Portugal. in *2020 IEEE Power & Energy Society Innovative Smart Grid Technologies Conference (ISGT)*, pp. 1–5, 10.1109/ISGT45199.2020.9087772 (IEEE, 2020).

[21] Gomes, E., Esteves, A., Morais, H. & Pereira, L. Leveraging explainable artificial intelligence in solar photovoltaic mappings: model explanations and feature selection. *Energies* **18**(5), 1282, 10.3390/en18051282 (2025).

[22] Pereira, L., Nair, V., Dias, B., Morais, H., and Annaswamy, A. Federated learning forecasting for strengthening grid reliability and enabling markets for resilience. in *IET Conference Proceedings* 2024, pp. 246–250, 10.1049/icp.2024.2608 (IET, Chicago, IL, USA, 2025).

[23] Quintal, F. *et al*. Energy monitoring in the wild: platform development and lessons learned from a real-world demonstrator. *Energies* **14**(18), 5786, 10.3390/en14185786 (2021).

[24] Copernicus Atmosphere Monitoring Service (CAMS), CAMS solar radiation time-series. Feb. (2020). Available at: https://ads.atmosphere.copernicus.eu/datasets/cams-solar-radiation-timeseries?tab=overview (Accessed: 13-Jan-2025).

[25] Anderson, K. S. *et al*. Pvlib python: 2023 project update. *Journal of Open Source Software* **8**(92), 5994, 10.21105/joss.05994 (2023).

[26] Martins, R. *Data-driven Modeling of Energy Consumption in Industrial Kitchens - Detection of Activations and Unsupervised Classification*, Available at: https://fenix.tecnico.ulisboa.pt/cursos/mecd/dissertacao/565303595503325 (M.Sc. thesis, Técnico Lisboa, University of Lisbon, Lisbon, Portugal, Nov. 2022).

